# Tyrosine phosphorylation of cortactin by the FAK-Src complex at focal adhesions regulates cell motility

**DOI:** 10.1186/1471-2121-12-49

**Published:** 2011-11-13

**Authors:** Wenqi Wang, Yang Liu, Kan Liao

**Affiliations:** 1 State Key Laboratory of Molecular Biology, Institute of Biochemistry and Cell Biology, Shanghai Institutes for Biological Sciences, Chinese Academy of Sciences, Shanghai 200031, China

**Keywords:** cortactin, cortactin tyrosine phosphorylation, FAK, FAK-Src complex, focal adhesions, cell motility

## Abstract

**Background:**

Cell migration plays an important role in many physiological and pathological processes, including immune cell chemotaxis and cancer metastasis. It is a coordinated process that involves dynamic changes in the actin cytoskeleton and its interplay with focal adhesions. At the leading edge of a migrating cell, it is the re-arrangement of actin and its attachment to focal adhesions that generates the driving force necessary for movement. However, the mechanisms involved in the attachment of actin filaments to focal adhesions are still not fully understood.

**Results:**

Signaling by the FAK-Src complex plays a crucial role in regulating the formation of protein complexes at focal adhesions to which the actin filaments are attached. Cortactin, an F-actin associated protein and a substrate of Src kinase, was found to interact with FAK through its SH3 domain and the C-terminal proline-rich regions of FAK. We found that the autophosphorylation of Tyr^397 ^in FAK, which is necessary for FAK activation, was not required for the interaction with cortactin, but was essential for the tyrosine phosphorylation of the associated cortactin. At focal adhesions, cortactin was phosphorylated at tyrosine residues known to be phosphorylated by Src. The tyrosine phosphorylation of cortactin and its ability to associate with the actin cytoskeleton were required in tandem for the regulation of cell motility. Cell motility could be inhibited by truncating the N-terminal F-actin binding domains of cortactin or by blocking tyrosine phosphorylation (Y421/466/475/482F mutation). In addition, the mutant cortactin phosphorylation mimic (Y421/466/475/482E) had a reduced ability to interact with FAK and promoted cell motility. The promotion of cell motility by the cortactin phosphorylation mimic could also be inhibited by truncating its N-terminal F-actin binding domains.

**Conclusions:**

Our results suggest that cortactin acts as a bridging molecule between actin filaments and focal adhesions. The cortactin N-terminus associates with F-actin, while its C-terminus interacts with focal adhesions. The tyrosine phosphorylation of cortactin by the FAK-Src complex modulates its interaction with FAK and increases its turnover at focal adhesions to promote cell motility.

## Background

Src is a non-receptor cytoplasmic tyrosine kinase activated by integrins and receptor tyrosine kinases [1]. In normal cells, Src is involved in a vast range of physiological functions, including cell proliferation, cytoskeletal regulation, cell shape control, cell-matrix adhesion dynamics and motility [2,3]. In many types of human cancer, Src is overexpressed or hyperactivated [4,5]. The prominent role of Src in regulating cytoskeletal dynamics and cell motility makes the study of Src indispensable in understanding cancer cell migration and invasion.

Initially identified as a tyrosine-phosphorylated protein in v-Src infected chicken embryo fibroblasts [6], cortactin is a direct substrate of cellular Src kinase [7]. It is phosphorylated by Src at three tyrosine residues (Tyr^421, 466, 482 ^of murine cortactin) *in vitro *[8]. The phosphorylation of Tyr^475 ^was identified by a mass spectrometry study [9]. These tyrosine phosphorylation sites reside in the proline-rich region, which is the least conserved domain in cortactin from different species [10].

Many studies have suggested that cortactin and its tyrosine phosphorylation regulate lamellipodial protrusion, cell spreading, intercellular adhesion and cell motility [11-13]. Src-catalyzed cortactin tyrosine phosphorylation is involved in integrin-mediated cell adhesion and spreading [14]. Cortactin knockdown in murine fibroblasts impairs both random and directional cell migration [15]. The expression of cortactin mutated at Src phosphorylation sites (Y421/466/482F) decreases cell motility in ECV304 endothelial cells [8]. The impaired cell motility in cortactin knockdown gastric cancer cell lines, with a low cortactin phosphorylation level, can be rescued by the ectopic expression of wild-type cortactin, but not by the mutant cortactin (Y421/466/482F) [16].

Early studies revealed that cortactin colocalizes with F-actin in the cortical structures of adherent cells [7,17]. It associates with the F-actin cytoskeleton through the F-actin binding tandem cortactin repeats and the N-terminal acidic domain that interacts with the actin-related protein (Arp) 2/3 complex for dendritic actin nucleation [10,18,19]. At the cell periphery, the F-actin cytoskeleton forms a highly organized meshwork that controls membrane protrusion and regulates cell motility [20,21]. During cell migration, the propelling force is generated by membrane protrusions and by membrane-matrix adhesions, called focal adhesions, at which transmembrane integrins link the extracellular matrix to the intracellular actin cytoskeleton [22].

In contrast to the cortactin that colocalizes with F-actin at cortical regions, tyrosine phosphorylated cortactin (pTyr^421, 466, 482^) is almost exclusively localized at focal adhesions [16,23]. It is colocalized with paxillin and vinculin at the ends of F-actin stress fibers [16,23]. At focal adhesions, the clustered integrins recruit FAK and facilitate its activation, forming an active FAK-Src complex that initiates many intracellular signaling events [24-27]. The autophosphorylation of FAK at Tyr^397 ^creates a high affinity binding site for the Src-homology 2 domain of Src kinase [28]. The binding of Src to FAK leads to the formation of an active FAK-Src complex in which the active Src kinase trans-phosphorylates FAK at Tyr^576, 577 ^for maximal FAK catalytic activity [29].

FAK recruits adaptor proteins and signaling molecules into focal adhesions, at which many are phosphorylated by the FAK-Src complex. The N-terminal FERM domain binds to growth factor receptors, the C-terminal FAT domain interacts with talin and paxillin, and the proline-rich regions recruit Src-homology 3 (SH3) domain-containing proteins, such as p130Cas, GRAF and ASAP1 [26,30].

In this study, we report that cortactin interacted with FAK at focal adhesions at which it is phosphorylated by the FAK-Src complex. Cortactin helps mediate the association of F-actin with focal adhesions. Its N-terminus interacts with F-actin, and its C-terminus associates with FAK. The tyrosine phosphorylation of cortactin by the FAK-Src complex reduces its interaction with FAK, most likely to increase its turnover at focal adhesions to promote cell motility.

## Results

### Colocalization of tyrosine phosphorylated cortactin and the active FAK-Src complex at focal adhesions

**Figure 1:**
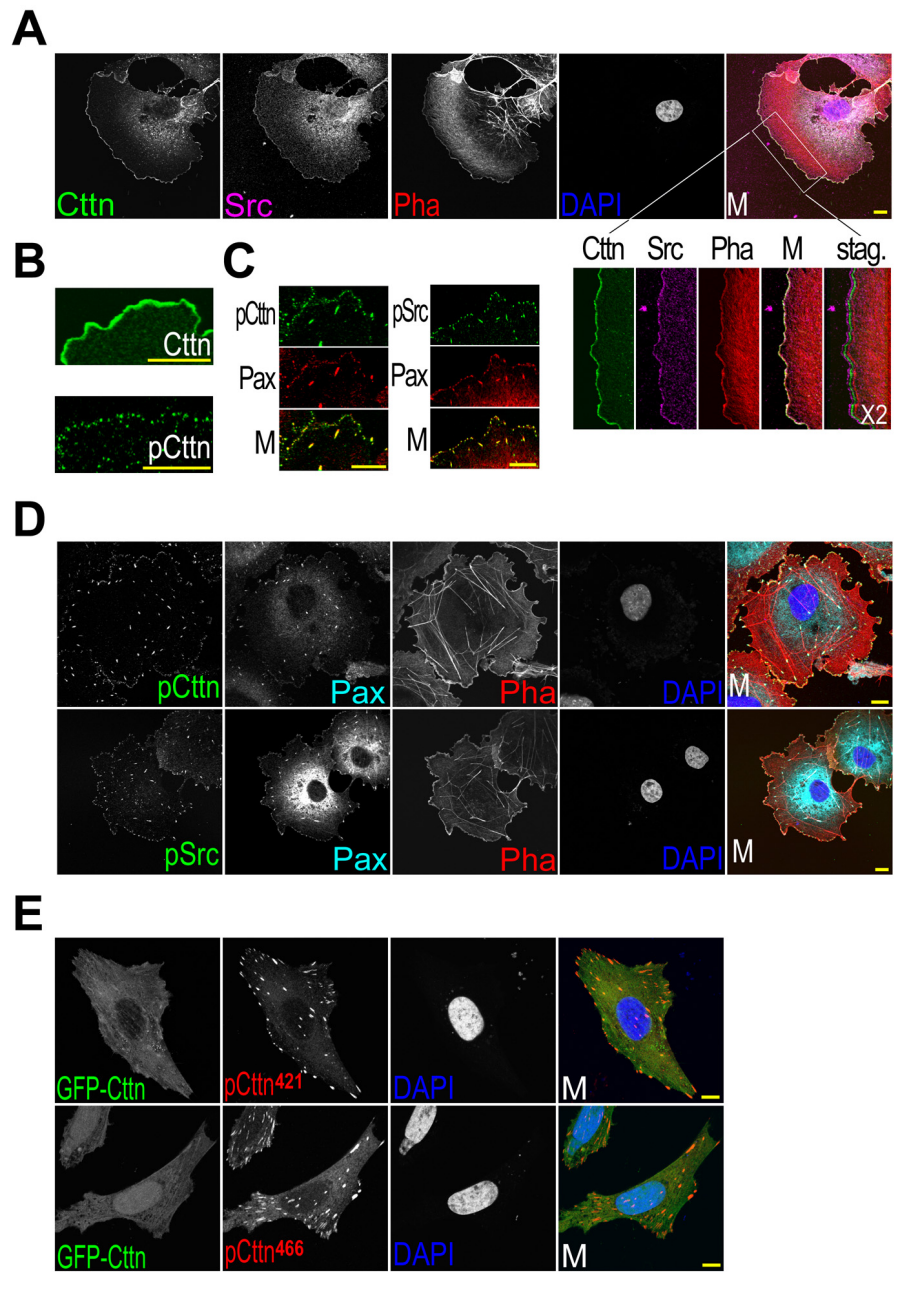
**Colocalization of tyrosine phosphorylated cortactin and active Src at focal adhesions**. Bar, 10 μm. (A) Colocalization of cortactin, Src and actin at the lamellipodial edge. COS7 cells were fixed with paraformaldehyde and Triton X-100, and stained with anti-cortactin antibody (*Cttn*), anti-Src antibody (*Src*), phalloidin (*pha*) and DAPI (*DAPI*). *M*, the merged picture; *stag*., the staggered picture. *X2*, 2 times enlargement. (B) The lamellipodial edge stained with an anti-cortactin (*Cttn*) or an anti-Tyr^421^-phosphorylated cortactin (*pCttn*) antibody. (C) Colocalization of Tyr^421^-phosphorylated cortactin (*pCttn*) and Tyr^418^-phosphorylated Src (*pSrc*) at focal contacts at the lamellipodial edge. *Pax*, anti-paxillin antibody staining. (D) Colocalization of Tyr^421^-phosphorylated cortactin and Tyr^418^-phosphorylated Src at focal adhesions. (E) The immunofluorescence staining of Tyr^421 or 466^-phosphorylated cortactin. COS7 cells expressing exogenous EGFP-tagged cortactin (*GFP-Cttn*) were stained with anti-phosphotyrosine^421 ^or anti-phosphotyrosine^466 ^antibody (*pCttn^421^, pCttn^466^*).

**Figure 2:**
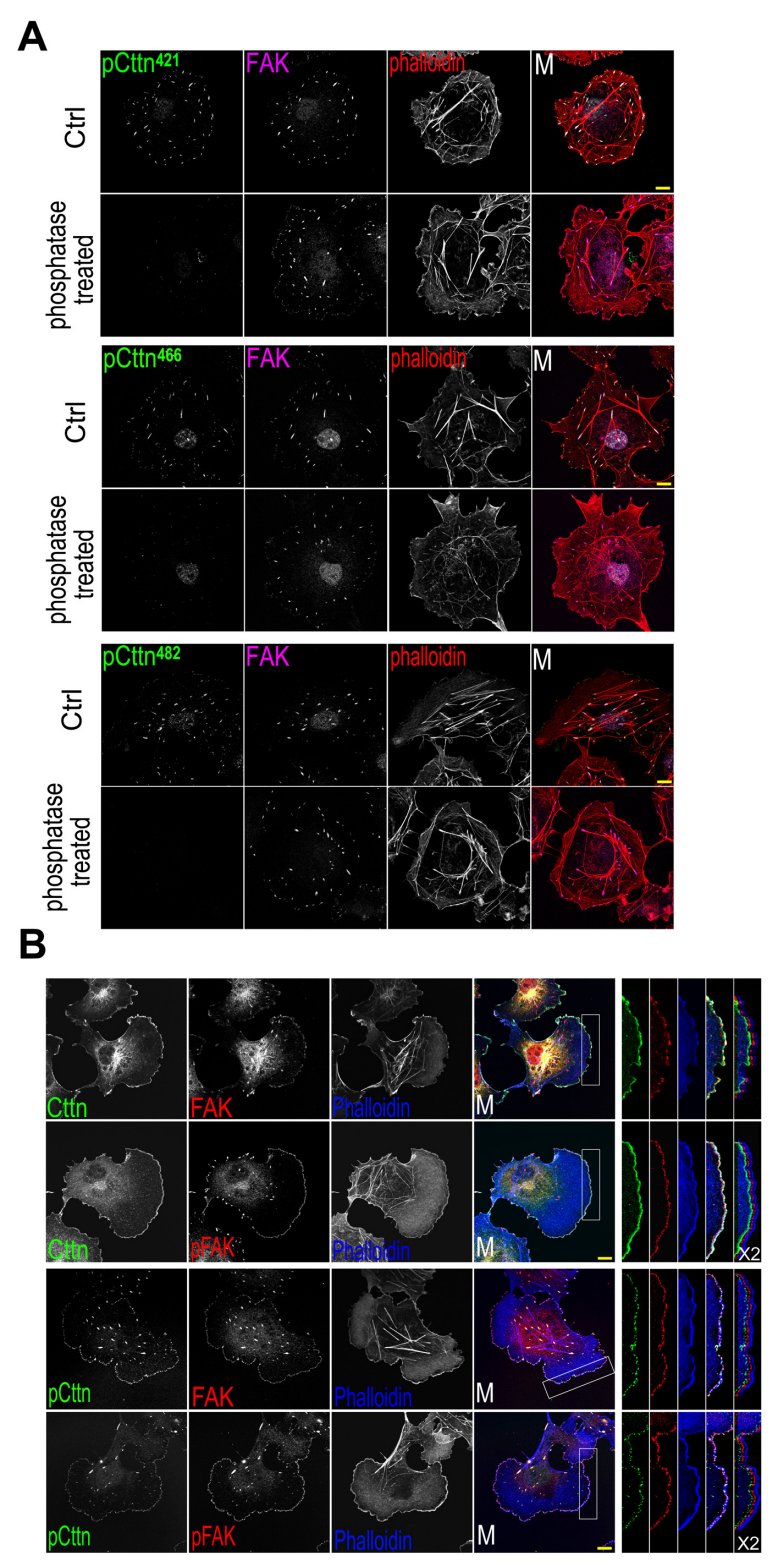
**Colocalization of tyrosine phosphorylated cortactin and FAK at focal adhesions**. Bar, 10 μm. (A) Dephosphorylation of Tyr^421, 466 or 482^-phosphorylated cortactin by alkaline phosphatase. Fixed COS7 cells were treated with 0.5 unit/μl calf intestinal alkaline phosphatase in 1 × TTBS (Tween/Tris-buffered saline, 25 mM Tris-HCl, pH 7.5, 150 mM NaCl, 0.05% Tween and 0.001% thimerosal) for 1 hour at room temperature, and then immunofluorescence-labeled with anti-FAK antibody (*FAK*), phalloidin (*phalloidin*), and with an anti-phosphotyrosine^421^, anti-phosphotyrosine^466 ^or anti-phosphotyrosine^482 ^cortactin antibody (*pCttn^421^, pCttn^466^, pCttn^482^*). *Ctrl*, without alkaline phosphatase treatment. (B) Colocalization of Tyr^421^-phosphorylated cortactin with FAK at focal adhesions. *FAK*, anti-FAK antibody staining. *pFAK*, anti-Tyr^397^-phosphorylated FAK antibody staining.

Cortactin, Src and actin are colocalized at the leading edge of lamellipodia in several types of cells, including COS7 cells (Figure 1A) [11]. In contrast, the tyrosine phosphorylated cortactin (at Src kinase site Tyr^421^) clustered into dots, which were localized to focal adhesions marked by paxillin (Figure 1D) [23]. The phosphorylated cortactin at focal adhesions was phosphorylated at all three Src kinase sites (Tyr^421, 466, 482^) (Figure 2A) (the information about these antibodies is provided in the Methods section). It could be dephosphorylated by alkaline phosphatase. No signal was detected by antibodies against phosphorylated cortactin in alkaline phosphatase-treated cells (Figure 2A). In addition, the active Src kinase (phosphorylated at Tyr^418^) was also colocalized with focal adhesions (Figure 1D). The kinase and its product of phosphorylated cortactin were colocalized at focal adhesions to which actin stress fibers were attached.

Cortactin and its phosphorylated forms had very different cellular distribution pattern in cell, implicating additional functions for phosphorylated cortactin (Figure 1E). At the lamellipodial leading edge, cortactin and its tyrosine phosphorylated forms had a different localization (Figure 1A and 1B). Cortactin traced the lamellipodial edge. However, phosphorylated cortactin dotted the edge and was colocalized with paxillin and Tyr^418^-phosphorylated Src at the newly formed focal contacts (Figure 1B and 1C). During cell-matrix interactions, the clustering of integrins forms focal contacts, and then focal adhesions, at the leading edge of the lamellipodia [31]. At focal adhesions, Src binds to Tyr^397^-phosphorylated FAK to form an active FAK-Src complex (Figure 1C and 1D) [26,28]. The colocalization of tyrosine phosphorylated cortactin with Tyr^418^-phosphorylated Src at focal adhesions implicates Src in cortactin tyrosine phosphorylation at focal adhesions (Figure 1D). It was further supported by the colocalization of phosphorylated cortactin with Tyr^397^-autophosphorylated FAK (Figure 2B).

### Cortactin interacts with FAK and its tyrosine phosphorylation reduces the interaction

**Figure 3:**
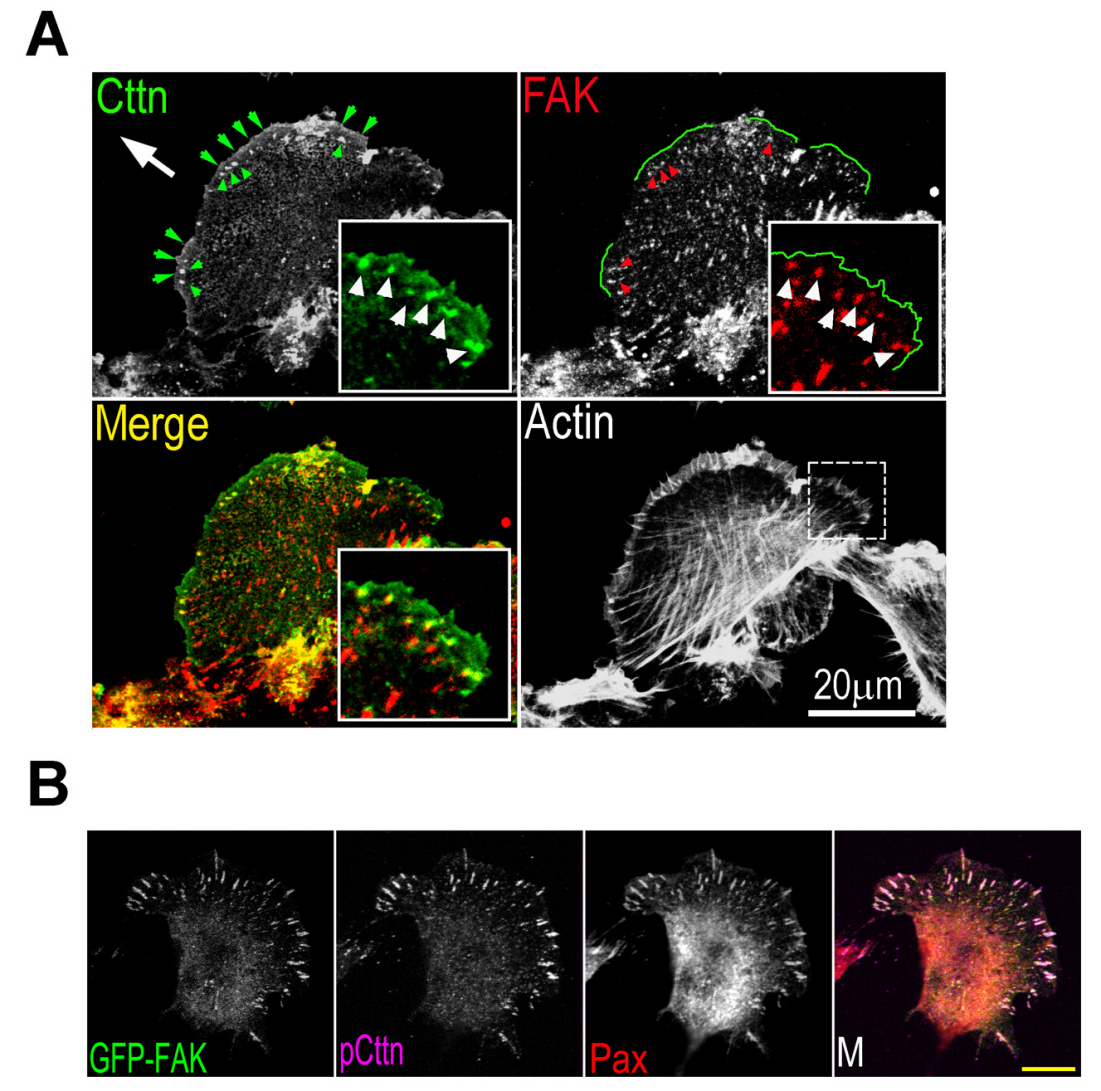
**Cortactin clustering at nascent focal adhesions in migrating cells**. (A) Immunofluorescence staining of cortactin and FAK on the leading edge of migrating HT1080 cells. Bar, 20 μm. The HT1080 cell monolayer was scratched with a rubber policeman. After 2 hours, the cells were fixed and stained with anti-cortactin antibody, anti-FAK antibody and phalloidin. The arrow indicates the direction of cell migration. The big arrowheads indicate the edge of lamellipodia, and the small arrowheads indicate focal adhesions. The insert represents the enlarged area. The images of the anti-cortactin and anti-FAK labeling are merged. (B) The localization of GFP-tagged FAK at focal adhesions. Bar, 20 μm. COS7 cells were transiently transfected with the GFP-tagged FAK expression vector and cells were fixed and stained with anti-cortactin (*Cttn*) and anti-paxillin (*Pax*) antibodies. *GFP-FAK*, GFP fluorescence. *M*, merged picture.

**Figure 4:**
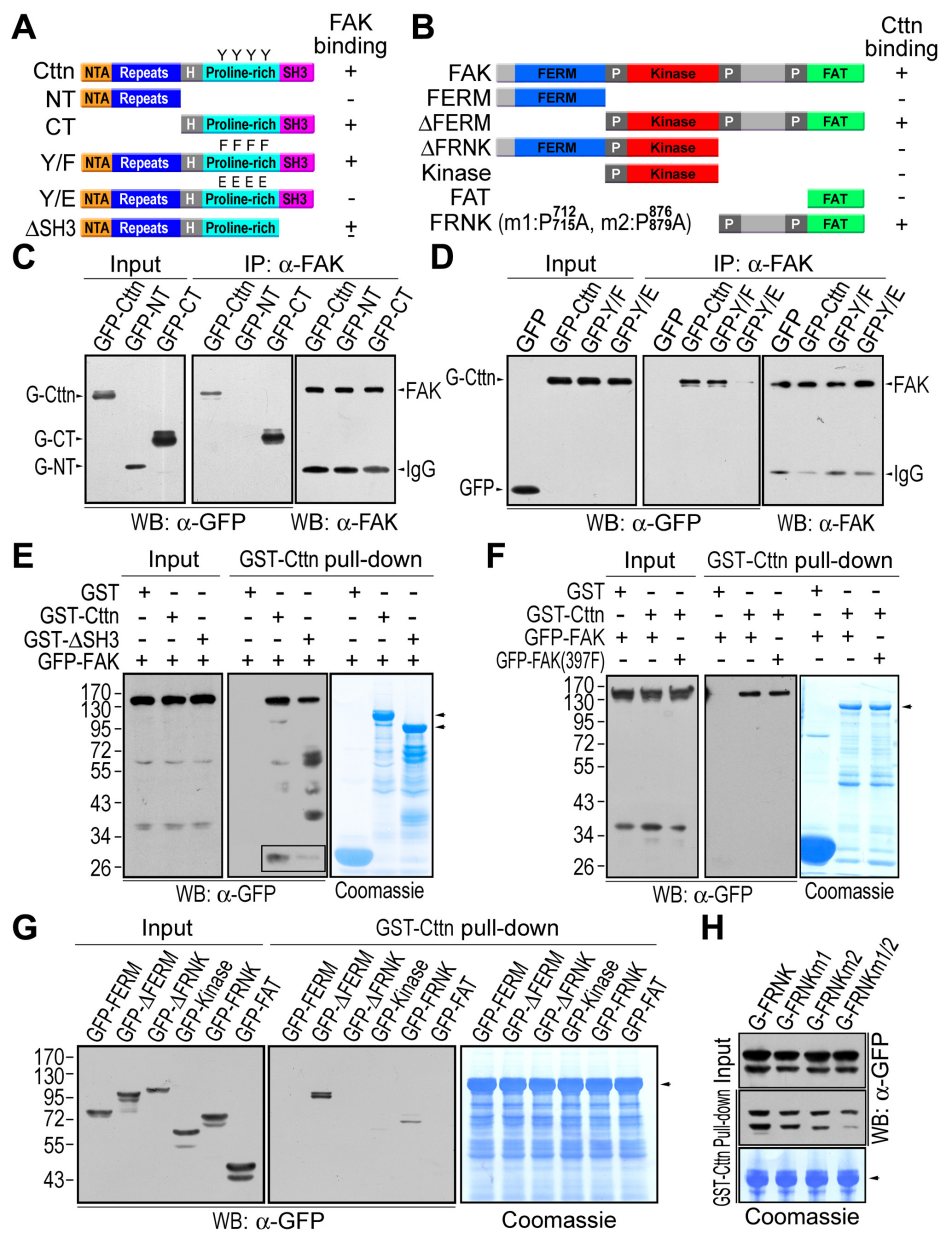
**Interaction of cortactin with FAK**. (A) Diagram of the various cortactin mutants. Y represents the tyrosine residues at amino acid positions 421, 466, 475 and 482. F and E indicate Phe and Glu mutations, respectively. *NTA*, the N-terminal acidic region; *Repeats*, the cortactin tandem repeats; *H*, the helix region; *Proline-rich*, the proline-rich region; *SH3*, the Src-homology 3 domain. (B) Diagram of the various FAK mutants. *P712/715A *and *P876/879A *indicate Ala mutations at Pro^712, 715 ^and Pro^876, 879^, respectively. *FERM*, the N-terminal FERM domain; *Kinase*, the central kinase domain; *FAT*, the C-terminal focal-adhesion targeting domain; *FRNK*, FAK-related non-kinase region; *P*, the proline-rich region. (C) GFP-tagged cortactin mutants expressed in COS7 cells were co-immunoprecipitated with anti-FAK antibody (*α-FAK*). *Input*, cell lysates; *WB*, western blot; *IP*, immunoprecipitation; *α-GFP*, anti-EGFP antibody. (D) The interaction of FAK with the cortactin phosphorylation mimic (Y/E) and phosphorylation-incompetent (Y/F) cortactin. (E) Interaction of SH3-deleted cortactin with FAK. GST fusion proteins immobilized on glutathione-beads were used to pull-down GFP-tagged FAK expressed in HEK293 cells. *Coomassie*, Coomassie blue staining. The arrow-heads indicate GST-cortactin and GST-SH3-deleted cortactin. The rectangle highlighted the low-exposed GFP-FAK bands. (F) The interaction of cortactin with FAK mutant (Y397F). GFP-tagged FAK or FAK mutant (Y397F) expressed in HEK293 cells was pulled-down with GST-cortactin. The arrowhead indicates GST-cortactin. (G) Interaction of cortactin with FAK proline-rich regions. GFP-tagged FAK mutants expressed in HEK293 cells were pulled down with GST-cortactin. (H) Interaction of cortactin with proline-rich region-mutated FRNK. *FRNKm1*, P712/715A FRNK; *FRNKm2*, P786/789A FRNK; *FRNKm1/2*, P712/715/786/789A FRNK.

Results from immunoprecipitation analysis indicated that cortactin interacts with FAK but not with paxillin (results not shown). Cortactin was clustered with FAK at newly formed focal adhesions at the leading lamellipodial edge of migrating cells (Figure 3A). To examine their interaction at focal adhesions, GFP-tagged cortactin mutants were expressed in COS7 cells, and the cellular extracts were immunoprecipitated using an anti-FAK antibody (Figure 4A). As shown in Figure 4C, only the cortactin C-terminus interacted with FAK. There was no interaction between the cortactin N-terminal F-actin binding domains and FAK.

The cortactin C-terminus contains two important functional domains: the proline-rich region containing Src kinase phosphorylation sites and the SH3 domain (7-10). The cortactin SH3 domain participates in the interaction with FAK. In GST pulldown experiments, the deletion of the SH3 domain reduced, but did not completely block, the interaction with FAK (Figure 4E). Cortactin tyrosine phosphorylation was not required for the interaction with FAK, but it regulated their interaction. The phosphorylation-incompetent cortactin (Y421/466/475/482F) bound FAK, similar to wild-type cortactin (Figure 4D). However, the cortactin phosphorylation mimic (Y421/466/475/482E) exhibited a much weaker association with FAK (Figure 4D). Thus, both the proline-rich region and the SH3 domain at the cortactin C-terminus were important for the cortactin-FAK interaction.

The autophosphorylation of FAK at Tyr^397 ^is essential for the binding to Src to form the FAK-Src complex. To determine if Src in the FAK-Src complex mediates the interaction between cortactin and FAK, Tyr^397 ^in FAK was mutated. Exogenous GFP-tagged FAK was expressed in COS7 cells and was found to localize to focal adhesions (Figure 3B). In the GST pull-down assay, the GFP-tagged FAK and the GFP-tagged FAK mutant (Y397F) exhibited no difference in their association with GST-cortactin (Figure 4F), indicating that the interaction between cortactin and FAK is not mediated by Src.

Of the several functional domains in FAK, the cortactin-interaction domain resides in proline-rich regions 2 and 3 (Figure 4B and 4G). By mutating the proline residues in these regions (m1 in proline-rich region 2 P712/715A, m2 in proline-rich region 3 P876/879A and m1/2 P712/715/876/879A), their interaction with cortactin was decreased (Figure 4H). This suggests that the interaction between cortactin and FAK is mediated by the C-terminal domains of cortactin and proline-rich regions 2 and 3 of FAK, and that it is regulated by cortactin tyrosine phosphorylation.

### Cell adhesion induced cortactin tyrosine phosphorylation at focal adhesions

**Figure 5:**
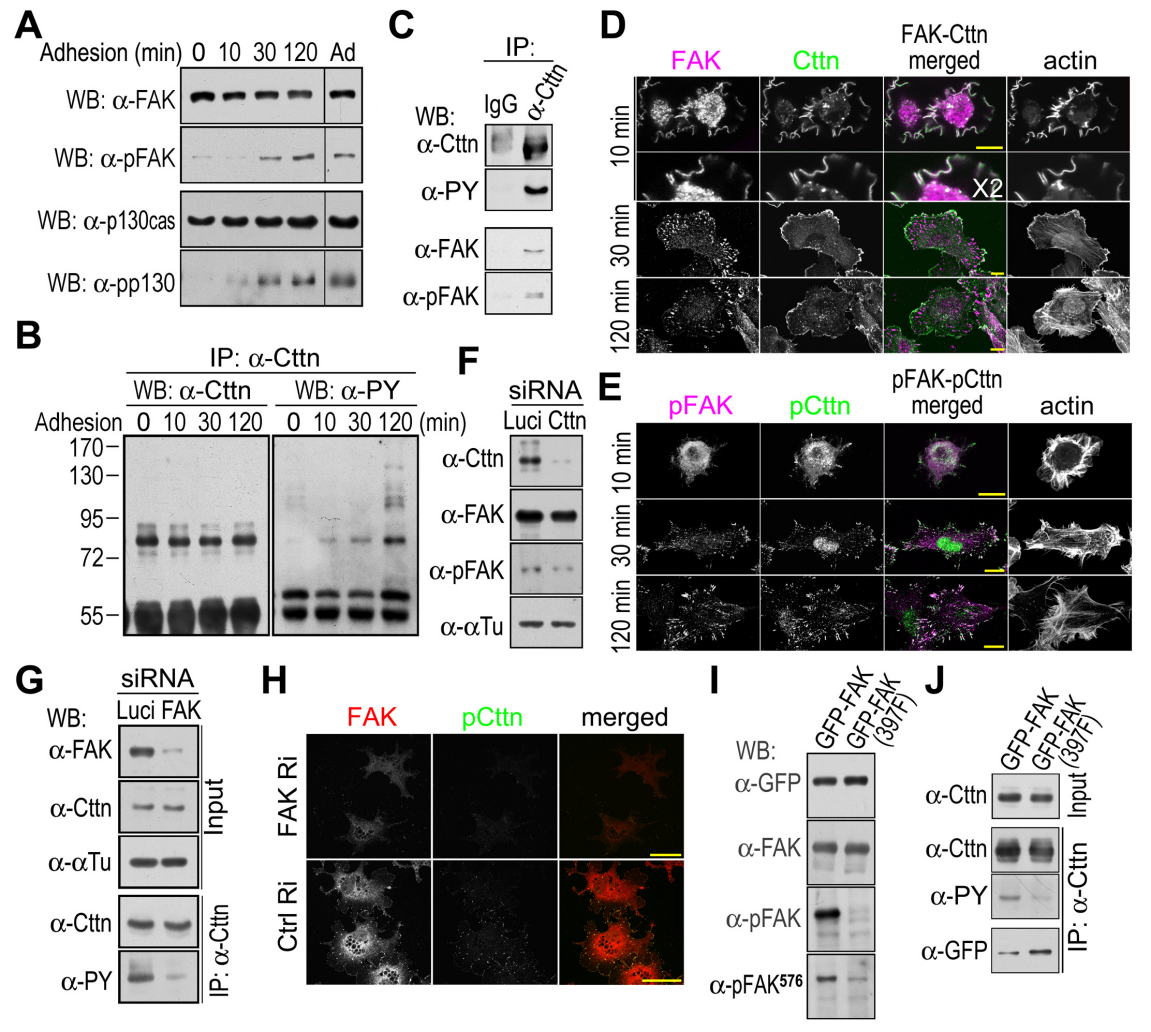
**Cell adhesion induced cortactin tyrosine phosphorylation requires FAK autophosphorylation at Tyr^397^**. (A) Adhesion induced FAK and p130Cas tyrosine phosphorylation. *10*, *30 *and *120 min *indicate the time elapsed after the HEK293 cells were plated onto fibronectin-coated dishes. *0 min*, suspended cells; *Ad*, cells cultured in dishes for 24 hours. *α-FAK*, *α-pFAK*, *α-p130Cas *and *α-pp130 *indicate western blots with anti-FAK, anti-phosphotyrosine^397 ^FAK, anti-p130Cas and anti-phosphotyrosine^410 ^p130Cas antibodies, respectively (B) Adhesion-induced cortactin tyrosine phosphorylation. Cortactin was immunoprecipitated (*IP*) from suspended HEK293 cells (*0 min*) and adhering cells (*10*, *30 *and *120 min*) with anti-cortactin antibody (*α-Cttn*). Cortactin tyrosine phosphorylation was detected with an anti-phosphotyrosine antibody (*α-PY*). (C) Co-immunoprecipitation of cortactin and FAK. Cortactin was immunoprecipitated from adherent HEK293 cells with anti-cortactin antibody (*α-Cttn*) or control IgG (*IgG*). The immunoprecipitated samples were analyzed by western blotting. (D) Immunofluorescence staining of FAK and cortactin during cell adhesion. Actin was stained with phalloidin. Bar, 10 μm. (E) Immunofluorescence staining of Tyr^397^-phosphorylated FAK and Tyr^421^-phosphorylated cortactin during cell adhesion. Bar, 10 μm. (F) FAK expression and autophosphorylation in cortactin knockdown cells. Cortactin was knocked down by RNA interference in COS7 cells. Cortactin, FAK and Tyr^397^-autophosphorylated FAK were detected by western blotting. α-Tubulin was used as the loading control. (G) Cortactin expression and tyrosine phosphorylation in FAK knockdown cells. FAK was knocked down by RNA interference in COS7 cells. *siRNA*, small interfering RNA; *Luci*, luciferase siRNA; *FAK*, FAK siRNA. FAK and cortactin proteins were detected by western blotting. Cortactin was immunoprecipitated with anti-cortactin antibody (*α-Cttn*) and its tyrosine phosphorylation was detected using an anti-phosphotyrosine antibody (*α-PY*). (H) Immunofluorescence staining of tyrosine phosphorylated cortactin in FAK knockdown cells. FAK RNAi (*FAK Ri*) and control RNAi (*Luci Ri*) cells were stained for FAK (*FAK*) and Tyr^421^-phosphorylated cortactin (*pCttn*). Bar, 50 μm. (I) Expression and phosphorylation of GFP-tagged FAK mutant (Y397F). The lysates of cells expressing GFP-tagged FAK or mutated FAK (Y397F) were blotted with anti-EGFP (*α-GFP*), anti-FAK (*α-FAK*), anti-phosphotyrosine^397 ^FAK (*α-pFAK*) or anti-phosphotyrosine^576 ^FAK (*α-pFAK^576^*) antibodies. (J) Cortactin tyrosine phosphorylation in cells expressing the GFP-tagged FAK mutant (Y397F). Cortactin in cells expressing the GFP-tagged or mutated (Y397F) FAK was immunoprecipitated with an anti-cortactin antibody and blotted with an anti-cortactin (*α-Cttn*), anti-phosphotyrosine (α-PY) or anti-EGFP (α-GFP) antibody.

Cell adhesion induces the formation of the FAK-Src complex, which leads to the phosphorylation of various FAK-associated proteins [26]. During the adhesion of suspended HEK293 cells in a fibronectin-coated dish, FAK autophosphorylation and the phosphorylation of FAK-associated p130Cas were both increased (Figure 5A). As a FAK associated protein, cortactin was also phosphorylated on tyrosine residues during this cell adhesion process (Figure 5B). The co-immunoprecipitation of FAK and its autophosphorylated form from adherent cells using an anti-cortactin antibody demonstrates the association of tyrosine phosphorylated cortactin with the FAK-Src complex (Figure 5C).

During the initial stage of cell adhesion and spreading, FAK, cortactin and actin were all colocalized at the edge of the membrane (Figure 5D). Cell adhesion induced FAK clustering and focal adhesion formation. FAK at focal adhesions was autophosphorylated (Figure 5D and 5E). In these adherent cells, cortactin was still primarily localized to the cell cortex, and cortactin clustering could only be observed at newly formed focal contacts at the leading edge of the lamellipodia (Figure 3A and 5D). However, cortactin was tyrosine phosphorylated at focal adhesions (Figure 2 and 5E). The tyrosine phosphorylation of cortactin might decrease its interaction with FAK, because the cortactin phosphorylation mimic (Y421/466/475/482E) exhibited a much weaker association with FAK (Figure 4D).

### FAK-Src complex is required for cortactin tyrosine phosphorylation at focal adhesions

To examine the function of the FAK-Src complex in cortactin tyrosine phosphorylation, FAK was knocked down by RNA interference in COS7 cells (Figure 5G and 5H). Cortactin expression was not affected by FAK knockdown, but cortactin tyrosine phosphorylation was greatly reduced (Figure 5G). Because focal adhesions can still form in FAK-deficient cells [32], cortactin tyrosine phosphorylation at focal adhesions in FAK knockdown COS7 cells was analyzed by immunofluorescence staining. As shown in Figure 5H, cortactin tyrosine phosphorylation was not detected at focal adhesions. In contrast, cortactin knockdown by RNA interference had no effect on FAK or its autophosphorylation (Figure 5F). Thus, these results suggest that at focal adhesions cortactin interacts with FAK, which is required for cortactin tyrosine phosphorylation.

FAK autophosphorylation at Tyr^397 ^is essential for the formation of the FAK-Src complex. By mutating Tyr^397 ^of FAK into Phe, the formation of the FAK-Src complex at focal adhesions can be disrupted [26]. In the GFP-tagged FAK (Y397F) mutant, autophosphorylation at Tyr^397 ^was abolished and Src-catalyzed Tyr^576 ^phosphorylation was inhibited (Figure 5I). However, the interaction of FAK with cortactin was not affected by the mutation at Tyr^397 ^in GST pull-down experiments (Figure 4F), and the Tyr^397^-mutated FAK, overexpressed in COS7 cells, could be co-immunoprecipitated with cortactin by the anti-cortactin antibody (Figure 5J). Although cortactin could still interact with the mutant FAK, the mutation disrupted the interaction between FAK and Src, leading to a decrease in cortactin phosphorylation. Tyrosine phosphorylation in cortactin immunoprecipitated from COS7 cells overexpressing the mutant GFP-tagged FAK (Y397F) was dramatically reduced (Figure 5J). Thus, it appears that cortactin tyrosine phosphorylation at focal adhesions requires the formation of the FAK-Src complex.

**Figure 6:**
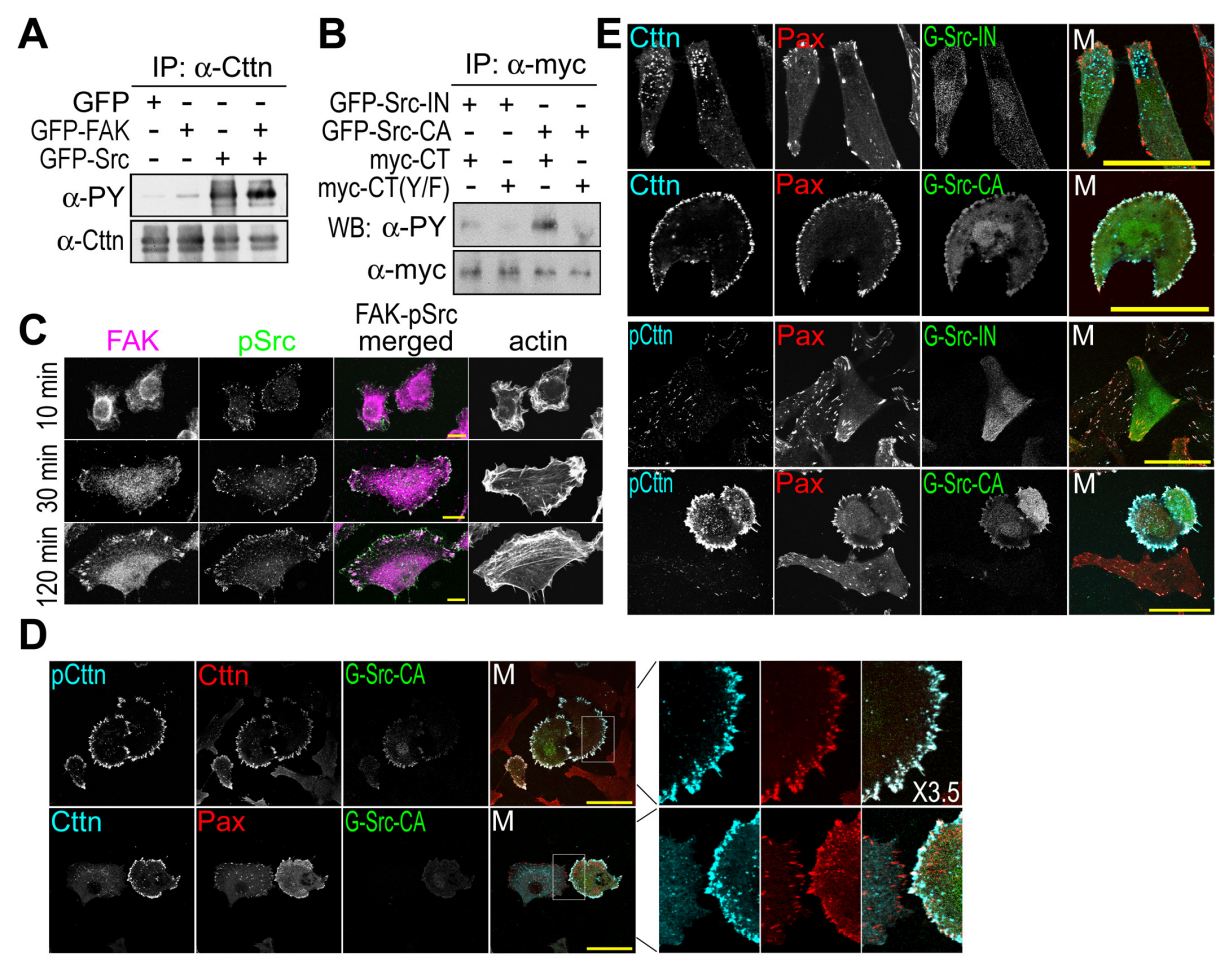
**Cortactin tyrosine phosphorylation by Src at focal adhesions**. (A) Enhanced cortactin tyrosine phosphorylation in cells overexpressing Src. Cortactin immunoprecipitated from HEK293 cells overexpressing GFP-tagged FAK, Src or both was blotted with an anti-phosphotyrosine antibody. (B) Incompetent cortactin tyrosine phosphorylation by mutating Src phosphorylation sites. Myc-tagged cortactin C-terminus (*myc-CT*) or mutated C-terminus (Y421/466/475/482F) [*myc-CT(Y/F)*] was co-expressed with GFP-tagged constitutively active Src (*GFP-Src-CA*) or inactive Src (*GFP-Src-IN*) in HEK293 cells and immunoprecipitated with anti-myc antibody (*α-myc*). The tyrosine phosphorylation of myc-tagged cortactin mutants was detected with anti-phosphotyrosine antibody (*α-PY*). (C) Activation of Src at focal adhesions during cell adhesion. Bar, 10 μm. *10*, *30 *and *120 min *indicate the time elapsed after the suspension of HEK293 cells were plated onto fibronectin-coated dishes. Cells were stained with anti-FAK and anti-phosphotyrosine^418 ^Src antibodies. Actin was stained with phalloidin. (D) and (E) Immunofluorescence staining of cortactin, Tyr^421 ^phosphorylated cortactin and paxillin in cells overexpressing GFP-tagged constitutively activated Src or inactive Src. Bar, 50 μm. NIH3T3 cells expressing GFP-tagged constitutively activated Src (*G-Src-CA*) or inactive Src (*G-Src-IN*) were stained with anti-cortactin (*Cttn*), anti-paxillin (*Pax*) and anti-phosphotyrosine^421 ^cortactin (pCttn) antibodies.

To determine whether cortactin is phosphorylated by FAK or by Src in the FAK-Src complex, cortactin tyrosine phosphorylation was analyzed in HEK293 cells overexpressing GFP-tagged FAK, Src or both. The results of this experiment suggest that cortactin was phosphorylated by Src (Figure 6A). Furthermore, cortactin tyrosine phosphorylation could be promoted by expressing the constitutively active Src, but not the inactive Src. And mutating the Src phosphorylation sites in cortactin (Y421/466/475/482F) inhibited Src-catalyzed cortactin tyrosine phosphorylation (Figure 6B).

The activation of Src in the FAK-Src complex was designated by an increase in Tyr^418^-phosphorylated Src following cell adhesion (results not shown). Src was recruited to focal adhesions by Tyr^397^-autophosphorylated FAK to form the FAK-Src complex during cell adhesion (Figure 6C). Although cortactin interacted with FAK at focal adhesions, it was phosphorylated by Src kinase in the FAK-Src complex. In cells overexpressing active Src, most cortactin was phosphorylated and recruited to cortical focal adhesions (Figure 6D and 6E). In these cells, cortactin and its phosphorylated form were well co-localized. In contrast, the expression of inactive Src did not affect cortactin phosphorylation or the formation of focal adhesions (Figure 6E).

### Cell motility is regulated by FAK-Src complex-catalyzed cortactin tyrosine phosphorylation

**Figure 7:**
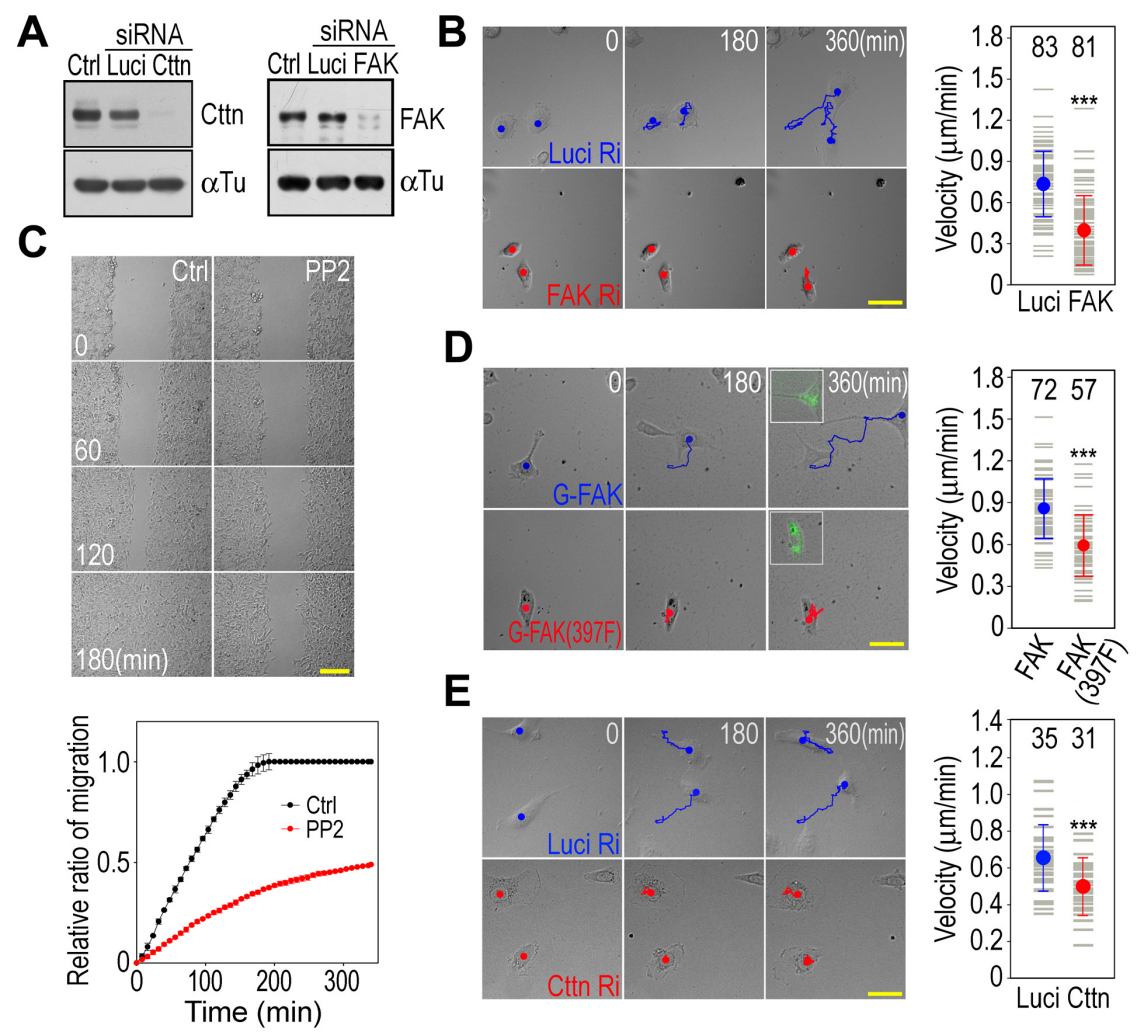
**Inhibition of cell migration by cortactin knockdown**. (A) Cortactin and FAK knockdown by RNA interference. Ctrl, HT1080 cells; Luci, luciferase siRNA control; Cttn, cortactin siRNA fragment; FAK, FAK siRNA fragment. Cortactin, FAK and α-tubulin were detected by western blotting. (B) Inhibition of cell migration by FAK knockdown. Bar, 50 μm. The track depicts the positions of the cells taken at 3-min intervals. The picture shows the cell at the starting point (*0*), at 180 min (*180*) and at 360 min (*360 min*). The plot is the results of 83 control RNAi cells (*Luci*) and 81 FAK RNAi cells (*Cttn*). ***P < 0.001. (C) Inhibition of cell migration by PP2. Bar, 200 μm. *Ctrl*, normal medium; *PP2*, medium with 10 μM PP2. Cell migration was determined by measuring the width of the gap at 4-minute intervals and normalized by the initial width of the gap. The averaged results of six wound-healing gaps at 8-minute intervals are plotted. (D) Inhibition of cell migration by overexpressing the FAK mutant (Y397F). Bar, 50 μm. GFP-tagged FAK or mutated FAK (Y397F) was expressed in HT1080 cells. The migration of the EGFP-positive cells was tracked. The inserts are EGFP fluorescence pictures of cells taken at 360 min. 72 GFP-FAK cells and 57 GFP-FAK (Y397F) cells were plotted. ***P < 0.001. (E) Inhibition of cell migration by cortactin knockdown. The labels are the same as in panel B.

The FAK-Src complex holds the center stage in the regulation of cell motility [26]. Cell migration is inhibited in FAK-deficient cells and in cells treated with the Src family kinase inhibitor, PP2 [32,33]. In HT1080 cells, FAK knockdown by RNA interference inhibited cell migration, as did inhibiting Src kinase activity with PP2 (Figure 7A, B and 7C). In addition, overexpressing the GFP-tagged FAK mutant (Y397F), which prevents formation of the FAK-Src complex, also inhibited cell migration (Figure 7D). As a substrate of the FAK-Src complex, cortactin was involved in regulating cell motility. Cortactin knockdown in HT1080 cells by RNA interference inhibited cell migration (Figure 7A and 7E).

**Figure 8:**
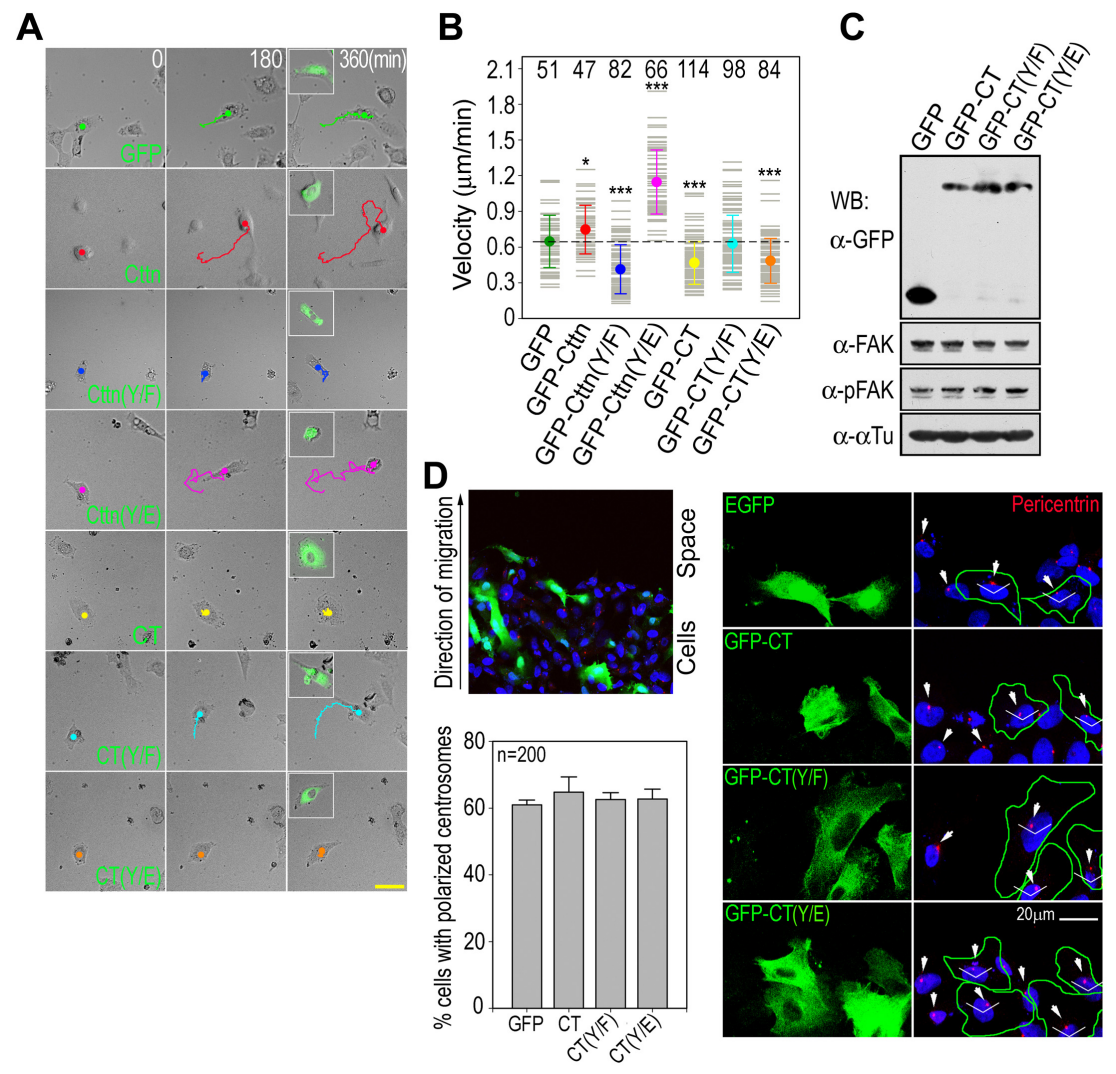
**Inhibition of cell migration by blocking cortactin tyrosine phosphorylation**. Tracks of live-cell recordings. Bar, 50 μm. The migration of HT1080 cells overexpressing EGFP (GFP), GFP-tagged cortactin (Cttn), the phosphorylation-incompetent cortactin mutant [Cttn(Y/F)], the cortactin phosphorylation mimic mutant [Cttn(Y/E)] or their N-terminal truncated forms [CT, CT(Y/*F*), *CT(Y/E)*] were tracked. The inserts are EGFP fluorescence picture of cells taken at 360 min. (B) Plot of migration speed. The numbers are the EGFP positive cells plotted. The statistical analysis was made against the results of cells overexpressing EGFP. *P < 0.05; ***P < 0.001. (C) FAK and its autophosphorylation in cells overexpressing N-terminal truncated cortactin mutants. FAK and its autophosphorylation at Tyr^397 ^in HT1080 cells overexpressing EGFP or GFP-tagged cortactin C-terminus mutants were analyzed by western blotting. (D) Centrosome orientation in migrating cells expressing GFP-tagged cortactin C-terminus mutants. HT1080 cells transfected with vector expressing GFP protein (*EGFP*), GFP-tagged cortactin C-terminus (*GFP-CT*), phosphorylation-incompetent C-terminus (Y421/466/475/482F) [*GFP-CT(Y/F)*] or phosphorylation mimicking C-terminus (Y421/466/475/482E) [*GFP-CT(Y/E)*] were subjected to wound-healing analysis. The centrosome (stained with anti-pericentrin antibody) in the GFP-positive cell at the migration front was analyzed. Cells with centrosomes located within 120° of the migration direction were counted as polarized, and 200 cells were plotted for each cortactin mutant.

Cortactin interacted with FAK and was phosphorylated by the FAK-Src complex (Figures 4 and 6). To investigate the role of FAK-Src complex-catalyzed cortactin tyrosine phosphorylation in cell motility, the phosphorylation sites in cortactin were mutated into Phe residues (Y421/466/475/482F) to block tyrosine phosphorylation. The Phe mutations in cortactin incompetent tyrosine phosphorylation by the FAK-Src complex, but did not block the interaction of cortactin with FAK (Figures 4D and 6B). Overexpressing the phosphorylation-incompetent cortactin mutant (Y421/466/475/482F) in HT1080 cells inhibited cell migration (Figure 8A and 8B), indicating that cortactin tyrosine phosphorylation regulates cell migration.

The tyrosine phosphorylation of cortactin by the FAK-Src complex most likely promotes its dissociation from FAK because the interaction between the cortactin phosphorylation mimic and FAK was greatly reduced (Figure 4D). The phosphorylation-incompetent cortactin mutant (Y421/466/475/482F) formed a stable interaction with FAK, and cell migration was inhibited. In contrast, the cortactin phosphorylation mimic exhibited increased turnover at focal adhesions (Figure 4D) [34], greatly enhancing cell migration (Figure 8A and 8B). Overexpressing wild-type cortactin in HT1080 cells led to an increase in cellular cortactin, which might increase levels of the phosphorylated form, thereby slightly enhancing cell migration (Figure 8A and 8B). Taken together, these results suggest that tyrosine phosphorylation of cortactin regulates cell motility by increasing cortactin turnover at focal adhesions.

Cortactin interacts with F-actin through its N-terminal F-actin binding domains [10]. In order to understand the effects of cortactin turnover on F-actin dynamics at focal adhesions, the cortactin N-terminal domains were truncated to disrupt its interaction with F-actin. Without these domains, the remaining cortactin C-terminus could still interact with FAK and could be phosphorylated by the FAK-Src complex (Figures 4C and 6B). However, cell migration was inhibited by overexpressing the cortactin C-terminus in HT1080 cells (Figure 8A and 8B). In addition, overexpressing the C-terminus of the cortactin phosphorylation mimic also inhibited cell migration (Figure 8A and 8B). Thus, the C-termini of cortactin and the phosphorylation mimic interfere with the ability of native cortactin to regulate cell migration.

The tyrosine phosphorylation of the cortactin C-terminus was essential for its inhibitory effect on cell migration. Cell migration was not inhibited by the phosphorylation-incompetent cortactin C-terminus (Figure 8A and 8B). The presence of the non-phosphorylated cortactin C-terminus did not affect the function of phosphorylated cortactin. In summary, the increased turnover of the cortactin phosphorylation mimic at focal adhesions increased F-actin dynamics and promoted cell migration. The stable association of the phosphorylation-incompetent cortactin at focal adhesions reduced F-actin dynamics and inhibited cell migration. Truncating the N-terminal F-actin binding domains did not affect F-actin dynamics. The stable association of the phosphorylation-incompetent cortactin C-terminus at focal adhesions appears to be independent of F-actin dynamics and then had no effect on cell motility. However, the mechanism by which the C-terminus of the cortactin phosphorylation mimic interferes with cellular phosphorylated cortactin and inhibits cell migration requires further study.

## Discussion

At focal adhesions, Src and FAK form an active kinase complex to phosphorylate associated proteins. The identification of cortactin, a Src kinase substrate and F-actin associated protein [10], as a FAK interactor at focal adhesions suggests that FAK-Src-catalyzed cortactin tyrosine phosphorylation is involved in the regulation of cell motility (Figure 4). Src was the first oncogene to be discovered and is one of the most intensely studied [1-3]. In cancer cells, active Src not only increases cell growth and survival, but also promotes actin cytoskeleton reorganization and decreases cell-cell and cell-matrix adhesions to facilitate motility and invasiveness [1,3]. Cortactin is frequently overexpressed in malignant tumors and there is a correlation between cortactin phosphorylation and enhanced cell migration and metastasis [35,36]. Cortactin and its tyrosine phosphorylation are involved in adhesion-dependent cell edge protrusion [37]. Cortactin is phosphorylated by Src at the leading edge of lamellipodia, where in association with the Arp2/3 complex, it initiates the branching of actin filaments to promote the formation of membrane protrusions [36,38]. The overexpression of constitutively active Src appears to induce cortactin phosphorylation and membrane protrusions at the edge (Figure 6D and 6E). Cell migration and invasion are essential elements of cancer metastasis. During cancer cell invasion, cortactin is one of the proteins that direct actin bundles to form invadopodia [39]. The involvement of Src-catalyzed cortactin phosphorylation in cell motility control suggests cortactin might be a crucial regulator of cancer metastasis.

For cell migration, the turnover of the cytoskeleton at focal adhesions is more important than stable association. To propel cell movement, the dynamic interplay between the actin cytoskeleton and cell adhesion sites induces membrane protrusions and generates traction force. The external force exerted on the cell and the internal force generated by the F-actin cytoskeleton during cell migration are anchored and sensed at focal adhesions, particularly by the FAK-Src complex [32,40]. One method of cell motility control by the FAK-Src complex is the phosphorylation of FAK associated proteins such as paxillin and p130Cas [40]. Mutating the FAK-Src phosphorylation sites in paxillin (Tyr^31, 118^) inhibits the turnover of focal adhesions and reduces cell motility [41]. Cortactin is phosphorylated by the FAK-Src complex at C-terminal tyrosine residues (Tyr^421, 466, 475, 482^; Figure 6) [8,9]. The structural configuration of cortactin, which contains N-terminal F-actin binding domains and C-terminal FAK association domains, enables it to mediate the association between the F-actin cytoskeleton and focal adhesions (Figure 4) [19]. By regulating the interaction between the cortactin C-terminus and FAK, cortactin tyrosine phosphorylation is able to control the association or dissociation of F-actin at focal adhesions. This hypothesis is supported by the results of the phosphorylation-incompetent cortactin mutant (Y421/466/475/482F) and its N-terminal domain truncated form. Blocking tyrosine phosphorylation by mutating Tyr into Phe (Y421/466/475/482F) stabilizes the interaction of cortactin with FAK, decreases the turnover of F-actin at focal adhesions and inhibits cell migration (Figure 4D and 8). By truncating the N-terminal F-actin binding domains, the remaining cortactin C-terminus can no longer interact with F-actin. Its association or dissociation at focal adhesions has no effect on F-actin dynamics--the inhibitory effect on cell migration by the stably-associated phosphorylation-incompetent cortactin could be reversed by truncating the N-terminal F-actin binding domains (Figure 8B).

The reduced interaction between the cortactin phosphorylation mimic and FAK suggests that cortactin dissociates from focal adhesions after being phosphorylated (Figure 4D). Our results and those of others indicate that the cortactin phosphorylation mimic promotes focal adhesion turnover and increases cell migration (Figure 8) [34]. It is very likely that cortactin tyrosine phosphorylation by the FAK-Src complex dissociates cortactin from focal adhesions and promotes F-actin turnover. The inhibition of cell migration by the N-terminal truncated cortactin phosphorylation mimic implies additional functions for tyrosine phosphorylated cortactin (Figure 8). Without the N-terminal F-actin binding domains, the phosphorylation mimic C-terminal can no longer interact with the F-actin cytoskeleton. This suggests that the C-terminus of the cortactin phosphorylation mimic interferes with the ability of cortactin to regulate motility. Importantly, the C-terminus of the phosphorylation-incompetent cortactin mutant does not have a similar inhibitory effect on cell migration (Figure 8). Thus, the effect of the cortactin phosphorylation mimic C-terminus on cell migration is phosphorylation specific. The wild-type cortactin C-terminus can also inhibit cell migration because it can be phosphorylated within the cell (Figure 6B and 8). It is not clear what functions are perturbed by the cortactin phosphorylation mimic C-terminus. FAK autophosphorylation and centrosome orientation, which are important for cell motility control, are not affected by the cortactin C-terminal mutants (Figure 8C and 8D). Further study is required to clarify the role of the cortactin phosphorylation mimic C-terminus on cell migration.

## Conclusions

In summary, we demonstrate that cortactin is recruited by FAK into focal adhesions, where it is phosphorylated by the FAK-Src complex. The interaction between FAK and cortactin is mediated by the SH3 domain and the proline-rich region and is regulated by FAK-Src complex-catalyzed cortactin tyrosine phosphorylation. Cortactin at focal adhesions can further recruit cortactin interacting proteins. The structure of cortactin, which contains N-terminal F-actin binding domains and C-terminal FAK association domains, allows it to function as a bridge between the F-actin cytoskeleton and focal adhesions. After the tyrosine phosphorylation of cortactin by the FAK-Src complex, the reduced interaction of cortactin with FAK leads to an increased turnover of the associated F-actin at focal adhesions. These changes in local F-actin dynamics ultimately regulate cell motility.

## Methods

### Materials

Anti-FAK, anti-phosphotyrosine^397 ^FAK, anti-Src, anti-phosphotyrosine^418 ^Src, anti-p130Cas and anti-phosphotyrosine^410 ^p130Cas antibodies were purchased from Cell Signaling (Danvers, MA, USA). Anti-phosphotyrosine^421 ^cortactin, anti-phosphotyrosine^466 ^cortactin, anti-α tubulin and anti-myc tag primary antibodies were from Sigma (St. Louis, MO, USA). DAPI, phalloidin, and horseradish peroxidase-conjugated secondary antibodies were also from Sigma. Anti-phosphotyrosine^482 ^cortactin antibody was from Chemicon. Anti-cortactin, anti-phosphotyrosine and anti-EGFP antibodies were from Santa Cruz Biotechnology (Santa Cruz, CA, USA). Anti-paxillin antibody was from Transduction Laboratories (San Jose, CA, USA). The fluorescence secondary antibodies (Alexa Fluor 488/546/647 conjugates) for immunofluorescence were from Invitrogen (Carlsbad, CA, USA). PP2 was from Calbiochem (San Diego, CA, USA). The Leica laser scanning confocal microsystem, including the Leica TCS SP2 confocal microscope, the Leica confocal scanner and the Leica confocal acquisition software, was used with the HCX PL APO 1bd. BL 63.0X/1.4 oil objective at 1.4 numerical aperture at a working temperature of 22°C. The fluorescence medium was Sigma's DABCO.

### Cell culture, immunofluorescence staining, immunoprecipitation and western blotting

COS7 cells, HEK293 cells, HT1080 cells and NIH3T3 cells were cultured in Dulbecco's modified Eagle's medium (DMEM) supplemented with 10% calf serum. For immunofluorescence staining, cells cultured on glass coverslips were fixed in 3.7% formaldehyde and 0.18% Triton X-100 in phosphate-buffered saline for 10 min. The fixed cells were stained with primary and secondary antibodies [42]. The detailed characterization on the specificity of the phospho-cortactin antibodies (anti-phosphotyrosine^421 ^cortactin antibody, anti-phosphotyrosine^466 ^cortactin antibody and anti-phosphotyrosine^482 ^cortactin antibody) is provided in the supplementary data of our previous publication [23]. For immunoprecipitation, cells were lysed in 1% Triton X-100 buffer containing 50 mM HEPES pH 7.4, 2.5 mM EDTA, 150 mM NaCl, 30 mM β-glycerophosphate, 1 mM sodium orthovanadate, 1 mM PMSF and 2 μl/ml protease inhibitor cocktail. After centrifugation at 12 000 g for 15 min at 4°C to remove cell debris, the supernatant (500-1000 μg protein) was mixed with 1.5 μg primary antibody for immunoprecipitation [43]. For western blotting, the sample in 1 × SDS sample buffer with 20 mM dithiothreitol was heated at 100°C for 5 min, subjected to SDS-PAGE and then transferred to an Immobilon-P membrane (Millipore, Billerica, MA). Western blotting was conducted as previously described [43]. The target proteins were detected using enhanced chemiluminescence.

### Construction and expression of cortactin, FAK and Src mutants and the analysis of their interactions

Murine cortactin cDNA, N-terminus (from aa 1 to 329), C-terminus (from aa 330 to 546), phosphorylation-incompetent mutant (Y421/466/475/482F), phosphorylation mimicking mutant (Y421/466/475/482E) and SH3-domain deletion mutant (from aa 1 to 500) were constructed into GFP-tagged, myc-tagged or GST-tagged expression vectors. Murine FAK cDNA, FERM (from aa 1 to 400), ΔFERM (from aa 401 to 1052), ΔFRNK (from aa 1 to 692), kinase domain (From aa 401 to 692), FRNK (from aa 693 to 1052), FAT (from aa 919 to 1052), FRNK m1 [FRNK mutant 1 (P712/715A)], FRNK m2 [FRNK mutant 2 (P876/879A)], FRNK m1/2 [FRNK mutant (P712/715/876/879A)] and the autophosphorylation-incompetent mutant (Y397F) were constructed into GFP-tagged expression vectors. The constitutively active Src (Y527F) and the inactive Src (K295M) were constructed from chicken Src. These tagged fusion proteins were expressed in cells by transient transfection following the protocol for Invitrogen's Lipofectamine 2000. The experiments were carried out 48 hours after transfection.

For co-immunoprecipitation, GFP-tagged fusion proteins were expressed in COS7 cells and the cell lysates were immunoprecipitated with anti-FAK antibody. For GST pull-down assays, GST-tagged cortactin was expressed in *E. coli *and isolated using glutathione-agarose beads. The lysates of HEK293 cells expressing GFP-tagged FAK mutants were incubated with GST-tagged cortactin bound to glutathione-agarose beads. The proteins were dissolved with 1 × SDS sample buffer and detected by western blotting.

### Cortactin tyrosine phosphorylation

HEK293 or HT1080 cells were resuspended in DMEM with 0.5% bovine serum albumin and plated onto culture dishes pretreated with 20 μg/ml fibronectin overnight and 1% Bovine serum albumin for 1 hour (Millipore-protocol). The cells were harvested at 10, 30 and 120 min for immunoprecipitation, immunofluorescence staining and western blotting. Suspended cells were considered 0 min, and normal cultured cells were adherent.

For RNA interference, COS7 cells were transfected with FAK stealth siRNA (5'-GGUUCAAGCUGGAUUAUUUTT-3'), cortactin stealth siRNA (5'-AUCUCUCUGUGACUCGUGCUUCUCCTT-3') or Luciferase control siRNA (5'-CGUACGCGGAAUACUUCGATT-3') at a final concentration of 80 nM following the protocol provided by Invitrogen. For expression of fusion proteins, COS7 and HEK293 cells were transfected with vector expressing GFP-tagged or myc-tagged fusion proteins. Forty-eight hours after transfection, cortactin tyrosine phosphorylation was analyzed.

### Cell migration and statistical analysis

In HT1080 cells, RNA interference was used to knock down cortactin or FAK. GFP-tagged proteins were expressed by transient transfection. Forty-eight hours after transfection, the cells were transferred onto plates with a glass bottom for live-cell microscopic imaging and cultured overnight. Cells were then transferred to Leibovitz's L15 medium with 10% FBS and mounted on a Leica MDW live-cell imaging station with a 37°C heated platform and a 20 × lens, and photographed every 3 min. The images were processed with Image J software on NIH Java and cells were tracked with Manual Tracking in Image J software. The position of the nuclear center for each cell was tracked, recorded and calculated for average migration speed in a 6-hour period. Eight view-fields were analyzed, and the cell numbers are indicated in the figures. For cortactin and FAK RNAi experiments, cells were randomly selected for analysis. For cells expressing GFP-tagged fusion proteins, only GFP positive cells were selected for analysis. Data are presented as mean ± SD. Differences were analyzed using Student's t-test. *P *< 0.05 was considered statistically significant.

The inhibition of cell migration by the Src family kinase inhibitor, PP2 [33], was assessed with the wound-healing assay. An HT1080 cell monolayer was scratched with a rubber policeman, washed with phosphate-buffer saline twice and fed with Leibovitz's L15 medium with 10% FBS in the presence or absence of 10 μM PP2. The cells were then mounted on the live-cell imaging station on a 37°C heated platform and 10 × lens and photographed every 4 min.

## Authors' contributions

WW performed plasmid construction, localization immunofluorescence, cell migration assays, immunoprecipitations and GST pull-downs. YL performed plasmid construction, localization immunofluorescence, cell transfections, protein phosphorylation analysis, immunoprecipitations and GST pull-downs. KL conceived of the study, participated in its design and coordination and drafted the manuscript. All authors read and approved the final manuscript.
